# Enhanced NADH Metabolism Involves Colistin-Induced Killing of *Bacillus subtilis* and *Paenibacillus polymyxa*

**DOI:** 10.3390/molecules24030387

**Published:** 2019-01-22

**Authors:** Zhiliang Yu, Yuyi Zhu, Jianv Fu, Juanping Qiu, Jianhua Yin

**Affiliations:** College of Biotechnology and Bioengineering, Zhejiang University of Technology, Hangzhou 310014, China; 2111405102@zjut.edu.cn (Y.Z.); 2111605001@zjut.edu.cn (J.F.); qiujping@zjut.edu.cn (J.Q.)

**Keywords:** colistin, Gram-positive bacteria, oxidative stress, TCA cycle, respiratory chain

## Abstract

The commonly believed mechanism of colistin against Gram-negative bacteria is to cause cell membrane lysis, whereas the mechanism of colistin against Gram-positive bacteria is extremely fragmented. In this study, we found that colistin treatment on *Bacillus subtilis* WB800, *Paenibacillus polymyxa* C12 and *Paenibacillus polymyxa* ATCC842 enhances not only the activities of α-ketoglutaric dehydrogenase and malate dehydrogenase in tricarboxylic acid (TCA) cycle, but also the relative expression levels of their encoding genes. Additionally, the oxaloacetate concentration also increases. Interestingly, the analysis of the relative expression of genes specific for respiratory chain showed that colistin treatment stimulates the respiratory chain in Gram-positive bacteria. Accordingly, the NAD^+^/NADH ratio increases and the oxidative level is then boosted up. As a result, the intensive oxidative damages are induced in Gram-positive bacteria and cells are killed. Notably, both rotenone and oligomycin, respectively, inhibiting NADH dehydrogenase and phosphorylation on respiratory chain can downgrade oxidative stress formation, thus alleviating the colistin-induced killing of Gram-positive cells. Besides, thiourea-based scavenging for reactive oxygen species also rescues the colistin-subjected cells. These data collectively demonstrate that colistin stimulates both TCA cycle and respiratory chain in Gram-positive bacteria, leading to the enhancement of NADH metabolism and resulting in the generation of oxidative damages in Gram-positive cells. Our studies provide a better understanding of antibacterial mechanism of colistin against Gram-positive bacteria, which is important for knowledge on bacterial resistance to colistin happening via the inhibition of respiratory chain and manipulation of its production.

## 1. Introduction

As a kind of alkaline polypeptide antibiotic produced by *Paenibacillus polymyxa*, polymyxin E, also called colistin, is increasingly used as one of the last-line therapeutic options for treatment on infections from multidrug-resistant Gram-negative bacteria [1,2,3]. The commonly believed mechanism of its action on the Gram-negatives is to cause cell membrane lysis [4]. Colistin can electrostatically interact with negatively charged lipopolysaccharide (LPS) and replace divalent cations on the outer membrane (OM) [5,6]. It will then penetrate OM via a self-promoted uptake mechanism and will result in the leakage of the inner membrane (IM), ultimately leading to cell death [7]. Yet, the action of colistin against Gram-positive bacteria has rarely been described [8,9]. Previous studies have shown that colistin is able to kill its producer *P. polymyxa*, a Gram-positive bacterium [10]. The proposed mechanism claimed that the positively charged residues on colistin may target the negatively charged residues of teichoic acids in the peptidoglycan sacculi, thereby enhancing cell membrane permeability and leading to cell death [10].

Recently, broad bactericidal antibiotics were found to induce the formation of reactive oxygen species (ROS), in particular the deleterious hydroxyl radicals (^•^OH). They can cause redox-related physiological alteration and toxic metabolic perturbation, ultimately resulting in cell death [11,12,13,14]. Accordingly, bactericidal antibiotics-induced ROS formation was proposed as a common mechanism of cell death, regardless of predominantly well-known drug-target interaction. This view has aroused much controversy [15,16]. Antibiotics not only act on specific target sites in cells, but they affect the overall metabolic network and physiologic status [17,18]. Studies have shown that antibiotics cause intracellular metabolic flow and flux changes in the tricarboxylic acid (TCA) cycle [19,20,21]. Antibiotics involve cell respiration [12,13] and they cause changes in the balance of intracellular iron, which is closely contracted to ROS production [22]. Besides direct lethality to cells, antibiotics-induced oxidative stress also causes a series of indirect damages, such as protein carbonylation, lipid peroxidation, and oxidative damage on DNA and RNA, which could also cause secondary damages [14]. Therefore, lethality that is caused by antibiotics is a complex physiological process [23]. The detail mechanism is believed to be more complex than what has been thought [14].

Very recently, we found that colistin can induce ROS accumulation in its producer *P. polymyxa* C12, a Gram-positive bacterium [24], regardless of cell membrane lysis [10]. However, the detail mechanism of oxidative stress formation by colistin is not clear yet. We highly expect that an understanding of the killing mechanisms of colistin against Gram-positive bacteria would not only extend our knowledge on antibacterial actions of colistin, but also benefit the manipulation of its production in the future. In this study, we demonstrate that colistin can induce oxidative stress in *B. subtilis* WB800 [25], *P. polymyxa* C12 [24], and *P. polymyxa* ATCC842 [26], leading to cell death. The generation of oxidative stress is due to sequenced stimulation of TCA cycle and respiratory chain, followed by the transient depletion of NADH.

## 2. Results

### 2.1. Oxidative Stress Caused by Colistin in Gram-Positive Bacteria

Our previous studies have shown that colistin can kill its producer *P. polymyxa* C12 [10,24]. In this study, minimal inhibitory concentration (MIC) of colistin against three Gram-positive bacteria was measured by disk diffusion assay. The results in Supplementary Figure S1 showed that MICs of colistin against WB800, C12 and ATCC842 were around 1 × 10^4^ U/mL, 8 × 10^4^ U/mL and 6 × 10^4^ U/mL, respectively, indicating that colistin has broad bactericidal activity to Gram-positive bacteria and *B. subtilis* is more sensitive to colistin than *P. polymyxa*.

**Figure 1:**
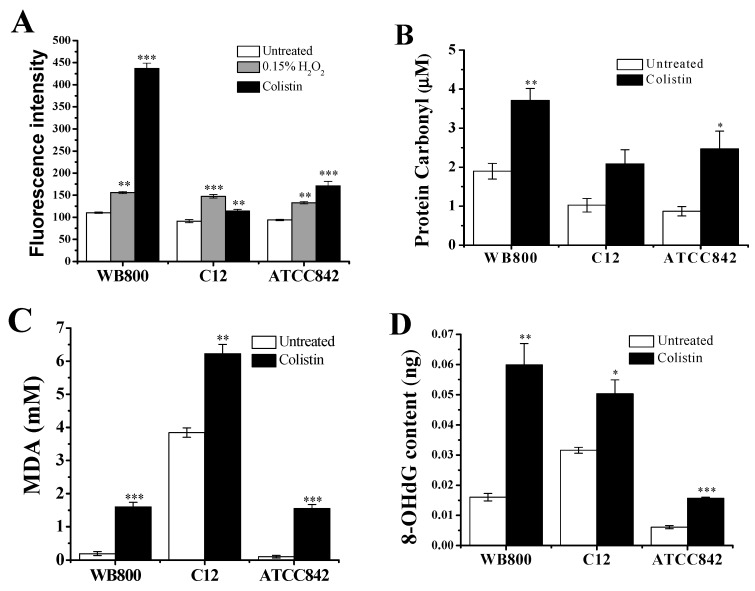
Colistin-induced oxidative stress in Gram-positive bacteria and damages to cells. (**A**) Hydroxyl radicals-specific hydroxyphenyl fluorescein (HPF) signal due to hydroxyl radicals accumulation; (**B**) protein carbonylation; (**C**) malondialdehyde (MDA) production; (**D**) 8-hydroxy-2-deoxyguanosine (8-OHdG) formation. Cells were treated with or without colistin for 2 h. The data are representative of three independent experiments. Points represent the means and bars represent the standard deviation of triplicate samples. When the probability (*p*-value) was less than 0.05, 0.01 and 0.001, the values were considered significantly (*), very significantly (**) and extremely significantly (***) different, respectively. WB800: *Bacillus subtilis* WB800; C12: *Paenibacillus polymyxa* C12; ATCC842: *Paenibacillus polymyxa* ATCC842.

It has been reported that killing of Gram-negative bacteria by colistin is mediated by ^•^OH formation [27]. We sought to determine whether colistin could also cause ^•^OH formation in Gram-positive bacteria. As positive control of 0.15% hydrogen peroxide (H_2_O_2_), colistin significantly increases the ^•^OH-trigged fluorescence intensity in Gram-positive bacteria: 250% increase in WB800, 23% increase in C12 and 82% increase in ATCC842 (Figure 1A), indicating that colistin does induce oxidative stress in Gram-positive bacteria.

^•^OH could result in broad oxidative damages including protein carbonylation, malondialdehyde (MDA) production and 8-hydroxy-2-deoxyguanosine (8-OHdG) formation in cells [28,29,30]. Figure 1B showed that the amount of carbonylated proteins in colistin-treated WB800, C12 and ATCC842 are increased by 96%, 104% and 184%, respectively, relative to the untreated control. In addition, Figure 1C indicated that colistin-treated WB800, C12 and ATCC842 have, respectively, 729%, 62% and 1422% increases in MDA content, relative to the untreated control. Figure 1D further showed that colistin-exposed WB800, C12 and ATCC842 yield 274%, 59% and 157% increases in 8-OHdG, respectively, relative to the untreated control. All of these data collectively demonstrate that colistin does result in oxidative damages that contribute to death of Gram-positive bacteria.

### 2.2. Scavenging Effect of Thiourea on Colistin-Induced Oxidative Stress

**Figure 2:**
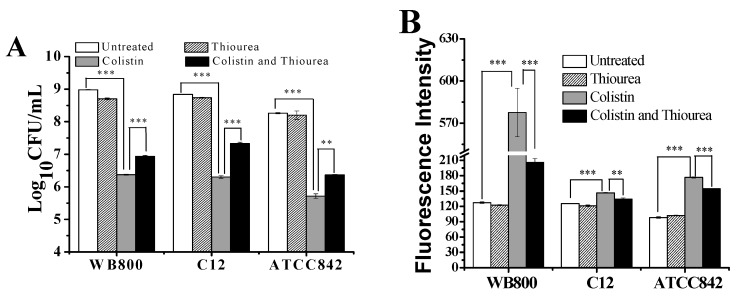
Saving of colistin-induced killing of Gram-positive bacteria by scavenging hydroxyl radicals with thiourea. (**A**) Changes in colony-forming units (CFU); (**B**) changes in hydroxyphenyl fluorescein (HPF) signal. Cells were treated with or without colistin for 2 h. The final concentration of thiourea for treatment was 150 mM. Data are representative of three independent experiments. Data were expressed as means and standard deviations. When the probability (*p*-value) was less than 0.01 and 0.001, the values were considered very significantly (**) and extremely significantly (***) different, respectively. WB800: *Bacillus subtilis* WB800; C12: *Paenibacillus polymyxa* C12; ATCC842: *Paenibacillus polymyxa* ATCC842.

Thiourea is a valid scavenger of ^•^OH [11]. Figure 2A showed that when compared to the untreated control, thiourea itself has no obvious effect on the colony-forming units (CFUs) of WB800, C12 and ATCC842. Conversely, colistin alone significantly decreases the CFUs of three Gram-positive bacteria by about three orders of magnitude. The addition of thiourea to colistin significantly restores the CFUs of WB800, C12 and ATCC842 by 0.56, 1.02 and 0.65 orders of magnitude, respectively. Figure 2B further showed that thiourea alone yields similar ^•^OH-trigged fluorescence intensity as the untreated control. On the contrary, colistin alone significantly enhances the fluorescence intensity in all three Gram-positive bacteria, indicating that colistin induces oxidative stress. When compared to colistin alone, colistin with thiourea significantly decreases the fluorescence intensities from 577 to 206 in WB800, from 146 to 134 in C12 and from 176 to 154 in ATCC842. Thiourea scavenging data further demonstrate that colistin can induce oxidative stress in Gram-positive bacteria and result in cell death.

### 2.3. Disturbance of TCA Cycle in Colistin-Exposed Gram-Positive Bacteria 

**Figure 3:**
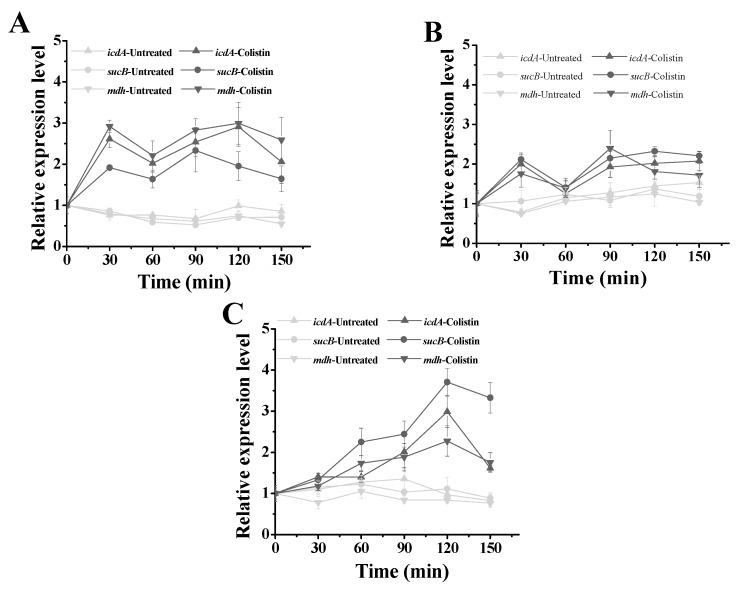
Effect of colistin on relative expression level of genes in the tricarboxylic acid (TCA) cycle. (**A**) *Bacillus subtilis* WB800; (**B**) *Paenibacillus polymyxa* C12; (**C**) *Paenibacillus polymyxa* ATCC842. *icdA* encoding isocitrate dehydrogenase; *sucB* encoding α-ketoglutaric dehydrogenase; *mdh* encoding malate dehydrogenase.

Studies have shown that flux changes in TCA cycle that are caused by antibiotics contribute to oxidative stress in Gram-negative bacteria [19,20,21]. However, it is not clear yet in Gram-positive bacteria. In the TCA cycle, isocitrate dehydrogenase (IcdA) encoded by *icdA* catalyzes the decarboxylation of isocitrate to produce α-ketoglutaric acid along with the conversion of NADP^+^ to NADPH. The α-ketoglutaric dehydrogenase (α-KGDH) encoded by *sucB* catalyzes the conversion of α-ketoglutarate to succinyl-CoA and malate dehydrogenase (MDH) encoded by *mdh* catalyzes the dehydrogenation of malic acid to form oxaloacetate along with the conversion of NAD^+^ to NADH. These three enzymes are important in TCA cycle, because they involve the supply of NAD(P)H for subsequent respiratory chain. To explore the disturbance of TCA cycle in Gram-positive bacteria exposed to colistin, the relative expression levels of *icdA*, *sucB* and *mdh* were detected. Figure 3 showed that the relative expression levels of all these three genes obviously increase in all three Gram-positive bacteria when exposed to colistin, as compared to the untreated controls, indicating that colistin could strengthen the TCA cycle in Gram-positive bacteria at the transcriptional level.

**Figure 4:**
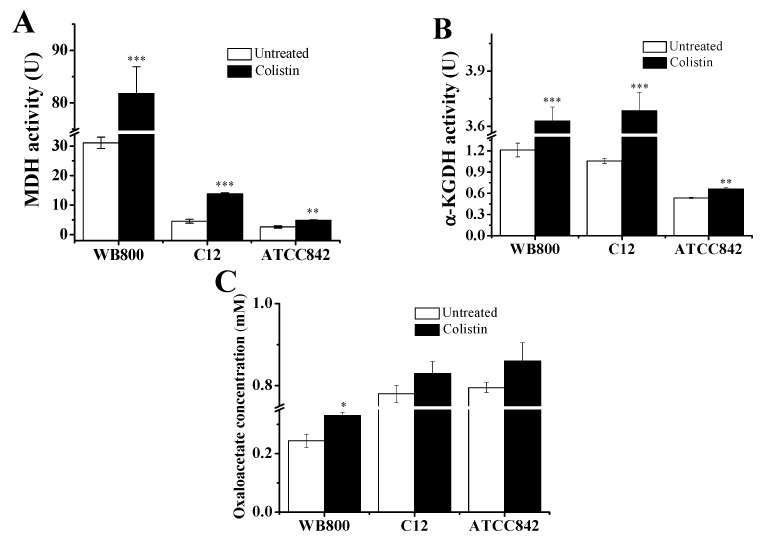
Colistin-induced changes in activity of enzymes and concentration of metabolite in TCA cycle. (**A**) Activity of malate dehydrogenase (MDH); (**B**) activity of α-ketoglutaric dehydrogenase (α-KGDH); (**C**) oxaloacetate concentration. Data were expressed as mean ± standard deviation. When the probability (*p*-value) was less than 0.05, 0.01 and 0.001, the values were considered significantly (*), very significantly (**) and extremely significantly (***) different, respectively. WB800: *Bacillus subtilis* WB800; C12: *Paenibacillus polymyxa* C12; ATCC842: *Paenibacillus polymyxa* ATCC842.

To further evaluate the stimulation of TCA cycle by colistin, the activities of MDH and α-KGDH together with oxaloacetate (the product of MDH) concentration were measured. Figure 4A showed that MDH activities are 81.8 U, 13.8 U and 4.86 U in colistin-treated WB800, C12 and ATCC842, respectively giving 163%, 204% and 85.9% increases relative to the untreated controls. Similarly, Figure 4B showed that α-KGDH activities are 3.63 U, 3.68 U and 0.66 U in colistin-treated WB800, C12 and ATCC842, respectively yielding 199%, 247% and 23.8% increases relative to the untreated controls. Figure 4C further showed that when compared to the untreated controls, the oxaloacetate concentrations in colistin-treated WB800, C12 and ATCC842 also increase. All of these findings further demonstrate that colistin strengthens the TCA cycle in Gram-positive bacteria.

### 2.4. Influence of Colistin on Respiratory Chain

**Figure 5:**
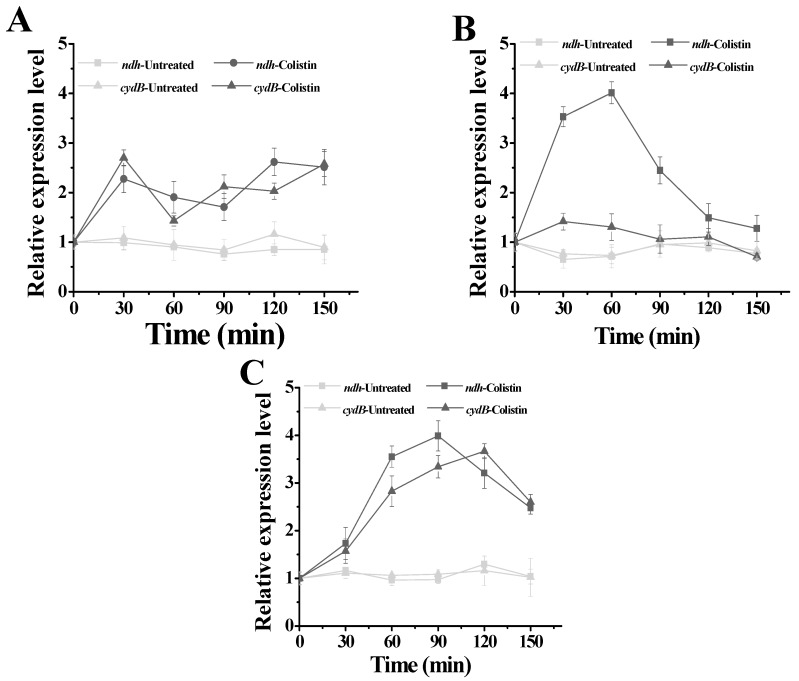
Effect of colistin on relative expression level of genes in respiratory chain. (**A**) *Bacillus subtilis* WB800; (**B**) *Paenibacillus polymyxa* C12; (**C**) *Paenibacillus polymyxa* ATCC842. *ndh* encoding NADH dehydrogenase; *cydB* encoding cytochrome *d* ubiquiton oxidase subunit 2.

**Figure 6:**
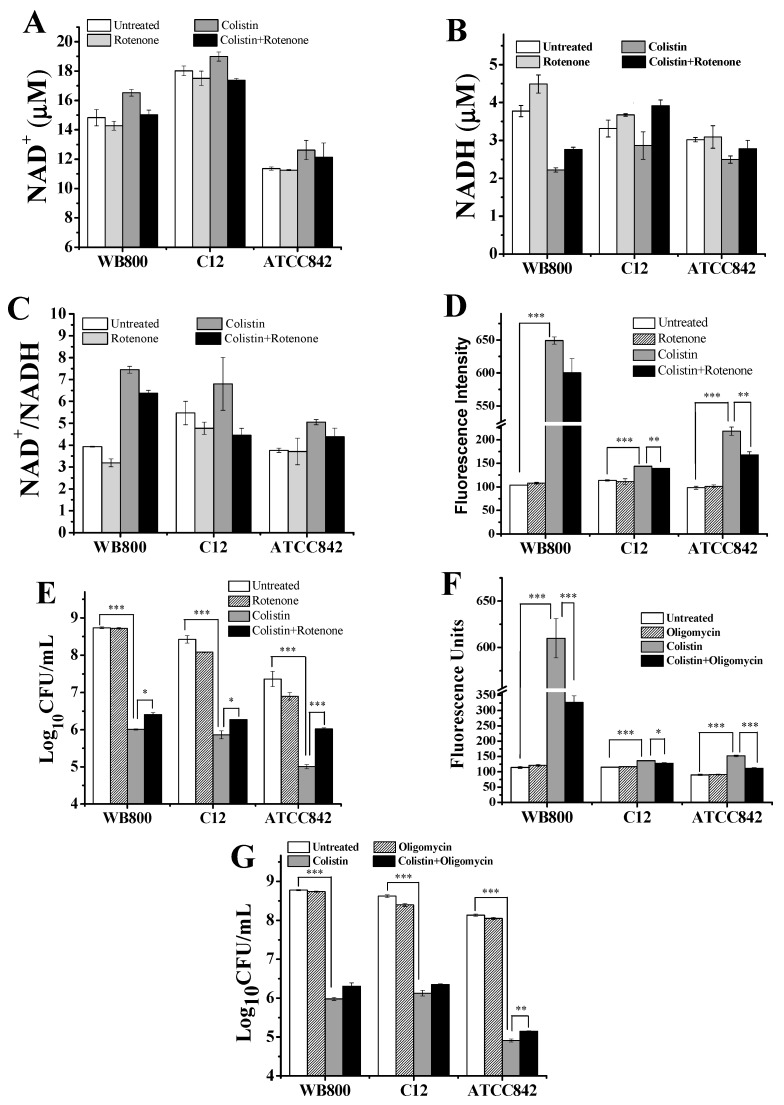
Enhanced NADH metabolism induced by colistin results in oxidative stress and killing of Gram-positive bacteria. (**A**) Changes in NAD^+^ content in cells when exposed to colistin with or without rotenone; (**B**) changes in NADH content in cells when exposed to colistin with or without rotenone; (**C**) changes in NAD^+^/NADH ratio in cells when exposed to colistin with or without rotenone; (**D**) changes in hydroxyphenyl fluorescein (HPF) signal resulted from hydroxyl radicals formation in cells when exposed to colistin with or without rotenone; (**E**) changes in colony-forming units (CFU) of cells when exposed to colistin with or without rotenone; (**F**) changes in HPF signal resulted from hydroxyl radicals formation in cells when exposed to colistin with or without oligomycin; (**G**) changes in CFU of cells when exposed to colistin with or without oligomycin. Colistin: 1 × 10^4^ U/mL, 8 × 10^4^ U/mL and 6 × 10^4^ U/mL for WB800, C12 and ATCC842, respectively; rotenone: 6 mg/L; oligomycin: 0.6 mg/L. When the probability (*p*-value) was less than 0.05, 0.01 and 0.001, the values were considered significantly (*), very significantly (**) and extremely significantly (***) different, respectively. WB800: *Bacillus subtilis* WB800; C12: *Paenibacillus polymyxa* C12; ATCC842: *Paenibacillus polymyxa* ATCC842.

NADH will be degraded by NADH dehydrogenase encoded by *ndh* in respiratory chain along with electron transport to release O_2_^−^ and form oxidative stress [31]. Both *B. subtilis* and *P. polymyxa* contain cytochrome *d*-type ubiquitone oxidases that are encoded by *cyd* which is important for electron transport in the respiratory chain [31]. In order to evaluate the effect of colistin on the respiratory chain at a transcriptional level, the relative expression levels of *ndh* and *cydB* (encoding cytochrome *d* ubiquiton oxidase subunit 2) were determined. As shown in Figure 5, colistin treatment clearly elevates the relative expression levels of both *ndh* and *cydB* in WB800, C12 and ATCC 842. Our findings indicate that colistin can stimulate the respiratory chain. As a result, the concentrations of NAD^+^ increase (Figure 6A), but the concentrations of NADH decrease (Figure 6B) in all three colistin-subjected strains. The increases in ratio of NAD^+^/NADH (Figure 6C) reveal that colistin could stimulate the conversion of NADH to NAD^+^. Accordingly, oxidative stress will be accentuated (Figure 1A). As an inhibitor of NADH dehydrogenase, rotenone can block electron transfer from NADH to CoQ in electron transport chain [32]. Our data showed that addition of rotenone to colistin can alleviate the colistin-induced increase of NAD^+^ concentration (Figure 6A) and decrease of NADH concentration (Figure 6B). Moreover, the addition of rotenone to colistin can reduce colistin-induced oxidative stress (Figure 6D) and rescue colistin-subjected cell survival (Figure 6E). Oligomycin is a phosphorylation inhibitor that binds to the oligomycin-sensitive protein which is F_0_ fraction of the F_0_F_1_-ATPase to block the hydrogen ion channels, thereby inhibiting the phosphorylation and ATP synthesis [33]. Figure 6F,G, respectively, showed that the addition of oligomycin can reduce colistin-induced oxidative stress and rescue colistin-subjected cell survival. These results collectively demonstrate that colistin strengthens the conversion of NADH to NAD^+^ along respiratory chain and results in oxidative stress accumulation, thus leading to cell death, which could be alleviated by respiratory inhibitors.

## 3. Discussion

Colistin is broadly used as the last-line drug for therapy of multidrug-resistant Gram-negative bacteria. Due to an impending crisis to treat infectious diseases that are induced by the emergence of antibiotic-resistant pathogens, urgent action of the specific sequence of events leading to cell death by colistin is needed for future drug advancement. Colistin is believed to kill Gram-negative bacteria through targeting negatively charged LPS and disrupting the membrane [4,5,6]. In contrast, the initiation target of colistin against Gram-positive bacteria is not very clear yet. Notably, recent studies indicated that colistin could induce the generation of harmful ROS in Gram-positive bacteria [27], which is consistent with the common death mechanism in the Gram-negatives caused by *β*-lactam, aminoglycoside and quinolone [13,34]. Yet, the mechanism of colistin-induced ROS generation in Gram-positive bacteria is not very clear.

**Figure 7:**
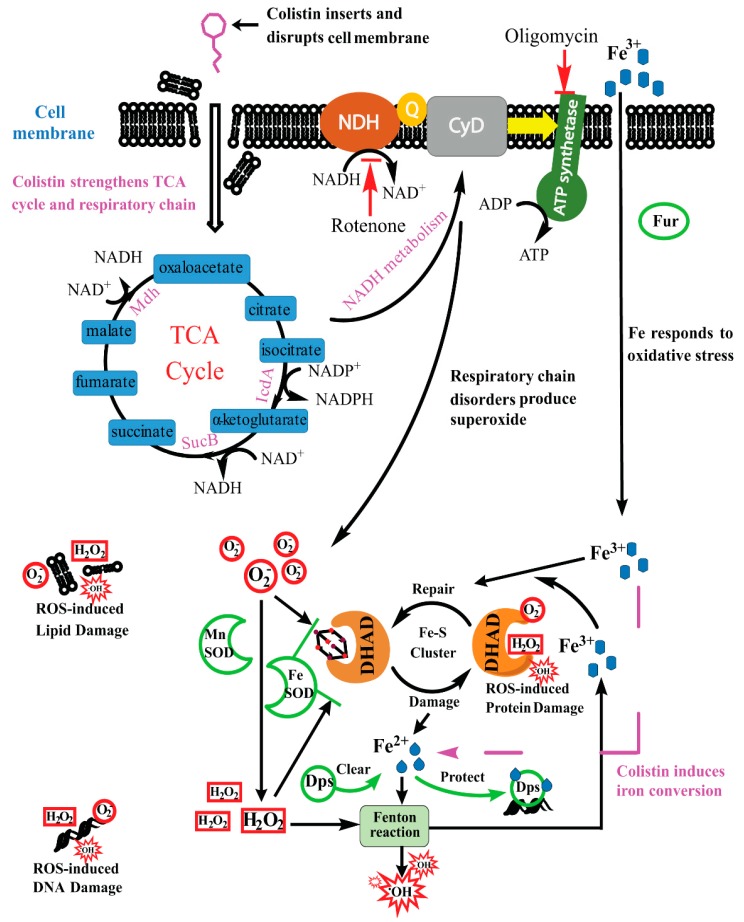
Proposed lethality of colistin against Gram-positive bacteria due to binary actions of cell permeability and oxidative stress [11,24,34]. DHAD: dihydroxy-acid dehydratase; Fur: ferric uptake regulator; Dps: DNA-binding protein from starved cells for sequestering Fe^2+^; NDH: NADH dehydrogenase; CyD: cytochrome *d*-type ubiquitone oxidases; SOD: superoxide dismutases; Fenton reaction: oxidization of Fe^2+^ by H_2_O_2_ to Fe^3+^ along with hydroxyl radicals (^•^OH) formation; O_2_^−^: superoxide; TCA cycle: tricarboxylic acid cycle; ROS: reactive oxygen species.

Our previous studies have demonstrated that colistin can disrupt the cell membrane of *P. polymyxa* [10], which then allows its act of entering inside (Figure 7). Subsequently, the TCA cycle is strengthened by colistin (Figure 4). In theory, NADH could be increasingly generated with the increase of activity of α-KGDH and MDH (Figure 4). In fact, colistin treatment results in the decrease of NADH content (Figure 6B). The probable reason is that the respiratory chain is also stimulated by colistin (Figure 5), which contributes to the accelerated consumption of NADH along the respiratory chain (Figure 6). The increase of ratio of NAD^+^ to NADH in colistin-subjected cells raises the speculation that the stimulation extent in the respiratory chain is stronger than that in TCA cycle and the depletion of NADH is transient [11]. Accordingly, the amount of O_2_^−^ will increase. In cells, O_2_^−^ will be converted to H_2_O_2_ by superoxide dismutase [10]. As a result, both O_2_^−^ and H_2_O_2_ can form the primary oxidative stress and attack Fe-S cluster with the release of Fe^2+^ (Figure 7). The burst of Fe^2+^ is a key source to drive the H_2_O_2_-involved Fenton reaction, generating very deleterious ^•^OH. When the concentration of ROS, particularly ^•^OH, reaches an uncontrollable level, DNA, lipids and proteins will be damaged (Figure 1), ultimately resulting in cell death. For survival, cells will accelerate Fe^3+^ assimilation and the ROS scavenging system will be boosted up for protection from oxidative damages [10].

Our data in Figure 6 indicated that rotenone and oligomycin respectively inhibiting NADH dehydrogenase and phosphorylation in the respiratory chain can decrease ROS generation, thus alleviating colistin-induced killing of the cells. Notably, there is evidence that some mechanisms of resistance to bactericidal drugs happen via inhibition of respiratory chain. This suggests that targeted inhibition of the respiratory chain may lead to increase in bactericidal drug resistance. On the other hand, both thiourea-based scavenging of ROS and the inhibition of respiratory chain cannot completely rescue the colistin-subjected cells. In addition, divalent cations neither give complete protection of *P. polymyxa* from colistin-induced membrane disruption [10]. All of these findings indicate that both cell permeability and oxidative damages contribute to bactericidal activity of colistin against the Gram-positives.

## 4. Materials and Methods

### 4.1. Strains and Growth Conditions

*P. polymyxa* C12 was supplied by Zhejiang Qianjiang Biochemical Co., Ltd. (Haining, China) and kept frozen at −80 °C in our lab [35,36]. *B. subtilis* WB800 and *P. polymyxa* ATCC842 were commercially purchased and also preserved in our lab. The medium for culture of C12 was prepared as below: beef exact 10 g/L, peptone 15 g/L, glucose 10 g/L, yeast extract 2 g/L, NaCl 3 g/L and FeSO_4_·7H_2_O 0.1 g/L. Both WB800 and ATCC842 were cultured in LB medium containing yeast extract 5 g/L, tryptone 10 g/L and NaCl 10 g/L. To make solid medium, agar was added to a final concentration of 20 g/L. Unless otherwise specified, *P. polymyxa* C12 was first grown on solid medium at 30 °C for two days. Subsequently, a ring of cells was inoculated to 50 mL of broth medium in 250 mL flask for incubation at 30 °C for 18 h with a shaking at 200 rpm. Similarly, both WB800 and ATCC842 were first grown on solid medium at 37 °C for one day. Afterwards, a ring of cells was transferred to 50 mL of broth medium in 250 mL flask for incubation at 37 °C for 12–16 h.

### 4.2. Treatment of Drugs on Strains

Upon broth incubation, the cells were collected by centrifugation at 8000 rpm for 10 min. After washing once with fresh broth medium, the cells were resuspended in fresh broth medium with appropriate volume to make a final cell concentration of around 10^9^ colony-forming units per milliliter (CFUs/mL). Unless otherwise specified, the cell solution of *P. polymyxa* C12 was treated by a final concentration of 8 × 10^4^ U/mL colistin without or with a final concentration of 0.15% H_2_O_2_, 150 mM thiourea, 6 mg/L rotenone or 0.6 mg/L oligomycin for 2 h at 37 °C with a shaking at 150 rpm. For *B. subtilis* WB800 and *P. polymyxa* ATCC842, colistin with 1 × 10^4^ U/mL and 6 × 10^4^ U/mL was respectively used. All of the drugs were purchased from Aladdin (Shanghai, China). One unit of colistin is equal to 0.0418 g.

### 4.3. Detection of Total Plate Count

After drug treatment, the mixture was centrifuged at 8000 rpm for 5 min. After washing once with fresh broth medium, the cells were resuspended and appropriately diluted in fresh broth medium. The 100 µL-volume of cells was then transferred to solid medium for growth. After cultivation for 1 d, the CFU was determined.

### 4.4. Measurement of Hydroxyl Radicals

Hydroxyl radicals-specific hydroxyphenyl fluorescein (HPF) was used to examine hydroxyl radicals [37]. After drug treatment, the mixture was centrifuged at 8000 rpm for 5 min. After washing once with fresh broth medium, the cells were resuspended in 1 mL of fresh broth medium and treated by HPF (Sigma, Beijing, China) with a final concentration of 5 M for 10 min. After washing once with fresh broth medium, the cells were resuspended in 1 mL of fresh broth medium. Fluorescence units in the cells were immediately measured by a multimode reader (SpectraMax M2, San Jose, CA, USA) with an excitation and emission of 490 nm and 515 nm, respectively.

### 4.5. Determination of Protein Carbonyl Content

The protein carbonyl content was determined by measuring the protein carbonyl residues using the dinitrophenylhydrazine (DNPH) [38]. In brief, the mixture was centrifuged at 8000 rpm for 5 min after drug treatment. Subsequently, the cells were collected and washed once with fresh broth medium. Next, the cells were resuspended in 700 µL of ddH_2_O and frozen at −80°C for 15 min, followed by water bath treatment at 100 °C for 15 min. After centrifugation at 8000 rpm 10 min at 4°C, the amount of protein-hydrozone product in the supernatant was measured using the Protein Carbonyl Colorimetric Assay Kit (Cayman Chemical, Ann Arbor, MI, USA), according to manufacturer’s instructions and quantified spectrophotometrically at the wavelength of 360 nm on a multimode reader (SpectraMax M2, San Jose, CA, USA).

### 4.6. Determination of Lipid Peroxidation

Lipid peroxidation was evaluated based on the production of malondialdehyde (MDA) which was quantified using thiobarbituric acid assay [24,39]. In brief, the mixture was centrifuged at 8000 rpm for 5 min after drug treatment. Afterwards, the cells were collected and washed once with fresh broth medium. Next, according to the lipid peroxidation assay kit (Beyotime, Jiangsu, China), the cells were resuspended in 100 μL of breaking solution containing 5% SDS and 250 mM EDTA for incubation at 37 °C for 30 min. Subsequently, 100 μL of MDA working solutions for each sample were prepared according to manufacturer’s instructions and added to cell solution. Next, the mixture was incubated at 100°C for 15 min. After centrifugation at 10,000 rpm for 10 min, the MDA in the supernatant was detected on a multimode reader (SpectraMax M2, San Jose, CA, USA) at a wavelength of 532 nm.

### 4.7. Detection of 8-OHdG Content 

After drug treatment, the mixture was centrifuged at 8000 rpm for 5 min and the cell pellet was washed once with fresh broth medium. Afterwards, the bacterial genome was extracted using EasyPure Bacteria Genomic DNA Kit (TransGen, Beijing, China). The amount of DNA for each sample can be 100 ng to 300 ng with good quality (OD260nm/OD280nm ratio >1.7 and no RNA contamination). According to the manufacturer’s instructions for EpiQuikTM 8-OHdG DNA Damage Quantification Direct Kit (Epigentek, Farmingdale, NY, USA), the standard curve was made and the 8-OHdG content in sample was determined.

### 4.8. Retrieval of Gene Sequences

After drug treatment, the mixture was centrifuged at 8000 rpm for 5 min and the cell pellet was washed once with fresh broth medium. Afterwards, the bacterial genome was extracted using EasyPure Bacteria Genomic DNA Kit (TransGen, Beijing, China). The primers that are listed in Table 1 and Table 2 were designed using Primer-BLAST software on NCBI according to the reference gene sequences from *B. subtilis* 168 (GenBank No. NC000964.3), *P. polymyxa* ATCC842 (GenBank No. NZGL905390) and *P. polymyxa* SC2 (GenBank No. NC014622.2). All of the primers were synthesized by Sangon Biotech (Shanghai, China). PCR amplification reactions were performed in a final volume of 50 µL containing 20 ng genomic DNA, 100 nM each of primers, 62.5 µM each of dNTPs, 50 mM KCl, 10 mM Tris-HCl, 1.5 mM MgCl_2_ and 1 U *Taq* polymerase (Amersham Biosciences, Piscataway, BJ, USA). PCR amplification consisted of denaturation at 95 °C for 5 min, followed by 35 cycles of 30 s at 95 °C, 30 s at 55 °C, 2 min at 72 °C, and a final extension step at 72 °C for 10 min. At the end of the reaction, the reaction mixture was cooled to 4 °C to await further manipulations. The PCR products were resolved on 1.0% agarose gel for electrophoresis, and the product size was checked on the gel that was stained with ethidium bromide under UV. After size confirmation, the target DNA in gel was extracted using MiniBEST Agarose Gel DNA Extraction Kit (TaKaRa, Dalian, China) and cloned into pMD19-T simple vector (TaKaRa, Dalian, China). Finally, each gene sequence was determined after sequencing (Sangon, Shanghai, China).

### 4.9. Analysis of Gene Expression Using Quantitative Real-Time PCR (qPCR)

To determine the gene expression, qPCR was used [40,41]. In brief, the bacterial cell was harvested and the total RNA was extracted using an RNAiso Plus (TaKaRa, Dalian, China). RNA integrity was evaluated based on the OD_260nm_/OD_280nm_ ratio, and 500 ng of DNA-free RNA with high-quality was reversely transcribed to cDNA in a 10 µL volume using PrimeScriptTM RT Master Mix (Perfect Real Time) kit. After proper dilution, the cDNA was applied for amplification of the target gene fragment with primer set (Table 3 and Table 4) by using the SYBR green Premix Ex TaqTM (Tli RNaseH Plus) kit. PCR was run on the CFX Connect Real-Time System (Bio-Rad, Hercules, CA, USA) with an amplification protocol consisting of an initial denaturation at 95 °C for 30 s, followed by 40 cycles of denaturation at 95 °C for 15 s and annealing/elongation at 60 °C for 30 s. A melting-curve analysis was immediately made to determine the reaction specificity based on the observed melting temperature from product. All of the operations were performed on ice condition.

The cycle threshold (*C*_T_) for each PCR was retrieved by using STATVIEW 5.0 (Informer Technologies, Inc., a software company made up of multinational programming teams, Los Angeles, CA, USA) which automatically sets the threshold signal at the log phase of amplification curve. Dilutions for each cDNA sample and each gene of interest were optimized in order to obtain a linear regression between the *C*_T_ value (ranging from 15 to 35 cycles) and the log of cDNA. The amplification efficiency for each gene was determined from the slope of that linear regression according to the formula E = 10^(−1/slope)^. The fragments of housekeeping genes 16S rRNA for *P. polymyxa* [24,36] and *sigA* for *B. subtilis* [42] were amplified and treated as the internal control to verify that there were equal amounts of target cDNA in all samples. The relative expression of the target gene as compared to that of the reference gene either 16S rRNA or *sigA* was calculated by the comparative *C*_T_ method [43].

### 4.10. Detection of MDH and α-KGDH Activities, and Oxaloacetate Concentration

After drug treatment, 1 mL of mixture was centrifuged at 8000 rpm for 5 min and the cell pellet was washed once with fresh broth medium. After collection, the cells were immediately transferred to ice. The activities of MDH and α-KGDH in cells were detected using Malate Dehydrogenase Assay Kit and α-Ketoglutarate Dehydrogenase Activity Colorimetric Assay Kit (Sigma, St. Louis, MO, USA), respectively, according to manufacturer’s instructions. The activities of MDH and α-KGDH in cells were determined based on the formation of NADH which was detected with absorbance at 450 nm. One unit of both MDH and α-KGDH is the amount of enzyme that generates 1.0 μmol of NADH per minute at 37 °C and pH 9.5. Oxaloacetate concentration in the sample was measured using the Oxaloacetate Assay Kit (Sigma, St. Louis, MO, USA) according to manufacturer’s instructions and determined based on fluorometric (Excitation/Emission = 535 nm/587 nm) product that is proportional to the oxaloacetate present.

### 4.11. Detection of NAD^+^ and NADH Levels

NAD^+^ and NADH contents were measured using the Amplite Colorimetric NAD/NADH Ratio Assay (AAT Bioquest, Sunnyvale, CA, USA) as reported previously [44]. After drug treatment, 100 μL of mixture was centrifuged at 13,000 rpm for 5 min and the cell pellet was washed once with fresh broth medium. After collection, the cells were dissolved into PBS buffer with lysozyme and proteinase K, and then incubated with shaking for 10 min at room temperature. The lysate was dispensed into two tubes for NADH and NAD^+^ measurements. For equilibrium adjustment, 300 μL of 0.2 M NaOH was added for NADH measurement, whereas 300 μL of 0.2 M HCl was added for NAD^+^ measurement, followed by incubation for 10 min at 50 °C. The sample was next kept on ice, and then 300 μL of 0.1 M NaOH and 300 μL of 0.1 M HCl were added for NADH and NAD^+^ measurement, respectively. After centrifugation at 13,000 rpm for 10 min, the supernatant was collected and then transferred to 600 μL of Bicine buffer. Finally, a 100-μL aliquot mixture was added to a 96-well plate and fluorescence units were immediately measured by a multimode reader (SpectraMax M2, San Jose, CA, USA) with an excitation and emission of 540 nm and 590 nm, respectively. The standard solutions of NADH with concentration from 0 to 10 μM were prepared and the standard curve between NADH concentration and fluorescence unit was made. The NADH and NAD^+^ levels in the sample were calculated using standard curve of NADH.

### 4.12. Data Analysis

Unless otherwise specified, triplicate reactions per experiment were performed. All data were expressed as mean ± standard deviation (SD) and tested for statistical significance based on analysis of variance (ANOVA), followed by Dunnetts post hoc test using the StatView5.0 program. When the probability (*p*-value) was less than 0.05, 0.01 and 0.001, the values were considered significantly, very significantly and extremely significantly different, respectively.

## 5. Conclusions

Data presented in this study demonstrated that colistin can stimulate TCA cycle and subsequently respiratory chain in three Gram-positive bacteria. As a result, the cycle of NADH metabolism will be prompted and oxidative stress will be accumulated, which results in intensive oxidative damages to cells. Gram-positive bacteria will accordingly be killed. Our studies would extend a better understanding of the antibacterial mechanism of colistin against Gram-positive bacteria, which is important in understanding bacterial resistance to colistin happening via the inhibition of respiratory chain. In addition, our knowledge on killing mechanisms of colistin against Gram-positive bacteria would benefit the manipulation of its production in the future.

## Figures and Tables

**Table 1:** Primers in PCR for *B. subtilis* WB800.

Genes	Nucleotide Sequences (5′–3′)		Product Size (bp)
	Forward	Reverse	
*icdA*	TCGCAACGACATCAAACT	CAACCCGATTATCCCATT	860
*sucB*	ATTCTTCCGCTTGAGTGC	GACGCCTGAATGTCTTGG	1465
*mdh*	GGTAGATGCCTTCATAGCC	AACCCGACAAAGGGAAAA	685
*ndh*	GCTGGTTATGGCGGAGTT	CAGTTGGCGGGTATGGAC	904
*cydB*	TACACGAGGGAATAGGGA	AGAACGCAGAGTGCTGAT	712

**Table 2:** Primers in PCR for *P. polymyxa*.

Genes	Nucleotide Sequences (5′–3′)		Product Size (bp)
	Forward	Reverse	
*icdA*	GTTGGAAGCCATCCGTGAGT	TGACGCCGGACAGAATTACG	866
*sucB*	CACAGGAACGCAAGTCGTTG	TACTCGAAGCCGAGGACTGA	639
*mdh*	CACCCAGATCATGGGCACTT	GTAGGCACACCGAGGAACAA	1462
*ndh*	GTGGATCGGATGCCCTTTCA	GTGGGCAATCTGCTCTCCTT	780
*cydB*	ACTGAGACGCTTATGGAAA	AAAGGGTGGACAGGAACG	666

**Table 3:** Primers in quantitative real-time PCR for *B. subtilis* WB800.

Genes	Nucleotide Sequences (5′–3′)		Product Size (bp)
	Forward	Reverse	
*icdA*	TCTTGTCTGAGCGCTACGTT	GTGGCTCCCTGCTGAAACAT	117
*sucB*	TCCATAGCGTCTGTACCCGA	GTGAATGCGGACGATCCTGA	114
*mdh*	ACCATATCGTCACCGTGTCC	AGGTGTGCTTGATACGGCAA	108
*ndh*	CACGCGTGACAGTGGTAAAC	TGGCATTGCAACCGCTTTTT	101
*cydB*	AACAGCGAGCGGAATGGTAA	CCGGAAAATGGCGCAAAAGA	124
*sigA*	GCCTGTCTGATCCACCACGTAGC	CGGTATGTCGGACGCGGTATG	137

**Table 4:** Primers in quantitative real-time PCR for *P. polymyxa.*

Genes	Nucleotide Sequences (5′–3′)		Product Size (bp)
	Forward	Reverse	
*icdA*	CCTATTGGCGGTGGTATCCG	TACTGGAGATGGGACACCGT	108
*sucB*	GCTTGTCCCTGTACTCGACG	TGCCAATACATTCAGACGGC	103
*mdh*	GGTACCGCTCGTACGCTATT	CACCCACTCGTGTACGTTGT	101
*ndh*	TCATCGCGCTAGGTTGTACC	CAAGCGCAGATACGTTTGGC	107
*cydB*	GCACTGTTGATCGAGCTCAC	GAAGGATGGGCTTTCGGGAT	104
16S rRNA	GAGAAGAAAGCCCCGGCTAA	ACCAGACTTAAAGAGCCGCC	116

## Supplementary Materials

Supplementary Figures S1 supporting this article is available online.
