# Supplementary material for: Enhanced NADH Metabolism Involves Colistin-Induced Killing of Bacillus subtilis and Paenibacillus polymyxa
Source: Molecules. 2019 Jan 22;24(3):387. doi: 10.3390/molecules24030387 (PMC6384706; doi:10.3390/molecules24030387)
Supplement: Supplementary file 1 [file molecules-24-00387-s001.pdf]

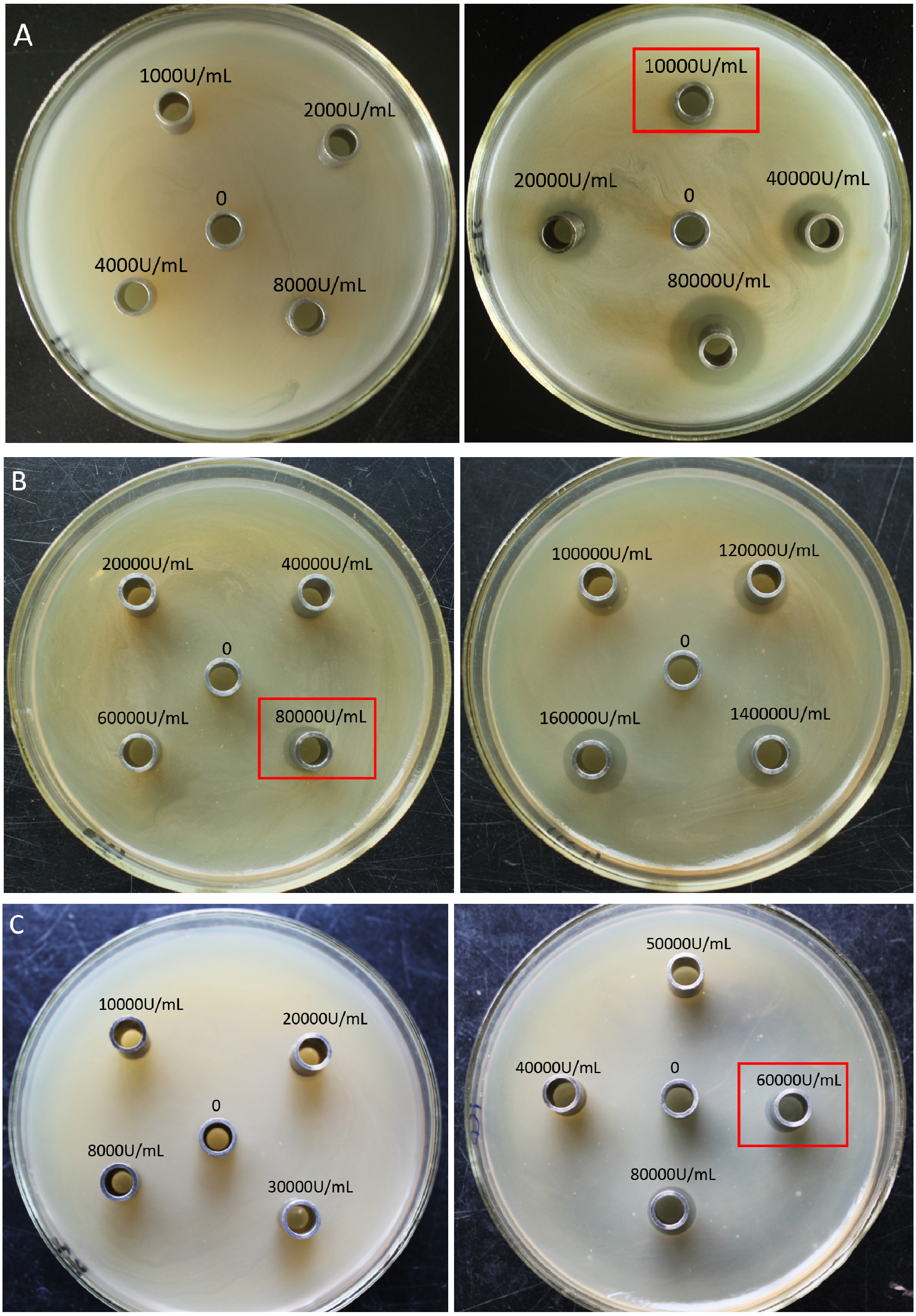

Supplementary Figure S1. Minimal inhibitory concentration (MIC) of colistin against three Gram-positive bacteria based on disk diffusion assay. (A) *Bacillus subtilis* WB800; (B) *Paenibacillus polymyxa* C12; (C) *Paenibacillus polymyxa* ATCC842.
